# Supplementary material for: Diet Quality Modulates Gut Microbiota Structure in Blastocystis-Colonised Individuals from Two Distinct Cohorts with Contrasting Sociodemographic Profiles
Source: Microorganisms. 2025 Aug 21;13(8):1949. doi: 10.3390/microorganisms13081949 (PMC12388865; doi:10.3390/microorganisms13081949)
Supplement: Supplementary file 1 [file microorganisms-13-01949-s001.zip › microorganisms-3783394-supplementary.pdf]

Table S1. Comparison of baseline characteristics and diet quality between the FACSA (university students) and PAVILA (institutionalised vulnerable child population) cohorts.

Variable	FACSA<br>N = 46[1]	PAVILA<br>N = 37[1]	p-value[2]
Age (years)			0.180
Mean ± SD	21.2 ± 1.4	17.9 ± 14.8	
Median (Q1, Q3)	21.0 (20.0, 22.0)	13.0 (7.0, 27.0)	
Weight (kg)			0.022
Mean ± SD	69.2 ± 15.8	57.2 ± 27.4	
Median (Q1, Q3)	67.5 (59.2, 77.8)	60.0 (32.9, 70.1)	
Height (m)			<0.001
Mean ± SD	1.7 ± 0.1	1.5 ± 0.2	
Median (Q1, Q3)	1.7 (1.6, 1.7)	1.6 (1.4, 1.6)	
BMI (kg/m^2)			0.471
Mean ± SD	25.2 ± 4.8	24.1 ± 7.9	
Median (Q1, Q3)	24.4 (22.2, 27.6)	23.2 (18.6, 26.9)	
Energy intake (kcal/day)			0.048
Mean ± SD	2,743.5 ± 907.9	3,338.0 ± 1,592.7	
Median (Q1, Q3)	2,651.5 (2,173.0, 3,055.0)	2,874.0 (2,282.0, 4,169.0)	
Dietary fiber (g/day)			0.055
Mean ± SD	72.0 ± 41.4	56.1 ± 32.6	
Median (Q1, Q3)	65.8 (51.0, 80.5)	54.6 (33.6, 65.0)	
HEI Score (0–100)			0.107
Mean ± SD	57.9 ± 9.5	54.1 ± 11.3	
Median (Q1, Q3)	59.8 (52.4, 63.6)	51.1 (47.0, 61.9)	
Qualitative diet quality			0.017
Good quality diet	1 (2.2%)	1 (2.7%)	
Intermediate quality diet	37 (80.4%)	19 (51.4%)	
Poor quality diet	8 (17.4%)	17 (45.9%)	
Physical activity level			0.751
Light	10 (21.7%)	8 (21.6%)	
Moderate	9 (19.6%)	5 (13.5%)	
Sedentary	27 (58.7%)	24 (64.9%)	

Sex			0.466
Male	14 (30.4%)	15 (40.5%)	
Female	32 (69.6%)	22 (59.5%)	

[1] n (%) for categorical variables

[2] Welch's Two-Sample t-test; Pearson's Chi-squared test as appropriate

We observed significant differences in weight (p = 0.022), height (p < 0.001), and energy intake (p = 0.048), with lower values for height and weight in PAVILA, but no differences in BMI. Despite apparently sufficient caloric intake, diet quality measured by the HEI-2020 was numerically lower in PAVILA (median 51.1) than in FACSA (median 59.8), although the difference was not statistically significant (p = 0.107). However, when qualitatively classifying the scores, the proportion of low-quality diets was significantly higher in the PAVILA cohort (45.9% vs. 17.4%; p = 0.017), suggesting a less healthy dietary pattern.

Table S2. Comparison of dietary quality scores between FACSA and PAVILA cohorts

Dietary Quality Variable	FACSA (n = 46)	PAVILA (n = 37)	p-value
Energy intake (kcal)	2651.50 [2174.75, 3053.25]	2874.00 [2282.00, 4169.00]	0.082
Caloric activity (kcal)	2275.00 [1959.00, 2802.25]	2044.00 [1771.00, 2457.00]	0.020
Water intake (g)	3382.73 [3058.83, 4101.15]	3577.86 [3043.08, 4362.50]	0.558
Fiber intake (g)	65.78 [51.13, 80.32]	54.61 [33.64, 65.04]	0.015
Total fruit score	3.20 [1.39, 4.78]	3.32 [3.02, 5.00]	0.325
Whole fruit score	0.75 [0.35, 1.86]	2.24 [0.67, 3.58]	0.024
Vegetables score	1.80 [1.50, 2.32]	1.48 [1.20, 1.96]	0.056
Legumes score	3.08 [2.56, 4.47]	3.37 [2.44, 5.00]	0.604
Whole grains score	10.00 [7.09, 10.00]	7.62 [3.49, 10.00]	0.028
Dairy score	3.39 [2.15, 4.48]	4.06 [2.50, 6.17]	0.156
Protein foods score	3.17 [2.99, 3.46]	2.87 [2.38, 3.43]	0.012
Seafood & plant proteins	3.09 [1.95, 5.00]	1.27 [0.00, 2.82]	<0.001
Fatty acid ratio score	3.92 [1.58, 4.98]	2.92 [0.23, 5.38]	0.527
Healthy fats score	8.98 [7.06, 10.00]	6.72 [4.07, 10.00]	0.027
Sodium score	10.00 [10.00, 10.00]	10.00 [10.00, 10.00]	0.950
Added sugars score	0.75 [0.00, 4.65]	0.00 [0.00, 0.10]	0.034
Saturated fat score	6.74 [4.83, 8.86]	6.33 [3.89, 8.01]	0.420
Total HEI-2020 score	59.75 [52.52, 63.48]	51.10 [47.00, 61.90]	0.036

Note: Values are expressed as median [Q1–Q3] for non-normally distributed variables.

Abbreviations: ft = total fruits; fw = whole fruits; ge = whole grains; prot = proteins; hr = healthy fats.

This table shows the specific differences in the HEI-2020 components between FACSA and PAVILA. We identified significantly lower scores in the PAVILA cohort for the following components: whole grains ($p = 0.028$), protein foods ($p = 0.012$), plant- or marine-based proteins ($p < 0.001$), healthy fats ($p = 0.027$), and higher intake of added sugars ($p = 0.034$). Furthermore, the PAVILA cohort had lower fibre intake ($p = 0.015$), reflecting lower dietary density. However, the PAVILA cohort had a significantly higher score on the whole fruit component (median: 2.24 vs. 0.75; $p = 0.024$). These differences reflect a significantly lower HEI-2020 total score in PAVILA (median: 51.10) compared to FACSA (median: 59.75; $p = 0.036$), suggesting lower overall dietary quality in the institutionalized population.

GA

Comparison of two Blastocystis-colonised cohorts: university students (FACSA) and institutionalised children and caregivers (PAVILA). Despite universal colonisation, gut microbiota diversity and composition differed markedly. FACSA showed higher diversity and abundance of Sellimonas, Murimonas, Alistipes, and Desulfovibrio, associated with better diet quality (higher intake of protein and vegetables). In contrast, PAVILA exhibited lower diversity, predominance of Hydrogenoanaerobacterium, V9D2013 group, and Haemophilus, and poorer diet quality. Age and social context also contributed to microbiota differences. Created with BioRender.com. Guangorena Gómez, J. (2025). Available at: https://BioRender.com/y9jgexd
